# Electronic faucet powered by low cost ceramic microbial fuel cells treating urine

**DOI:** 10.1016/j.jpowsour.2021.230004

**Published:** 2021-09-15

**Authors:** Irene Merino Jimenez, Patrick Brinson, John Greenman, Ioannis Ieropoulos

**Affiliations:** a Bristol BioEnergy Centre, Bristol Robotics Laboratory, University of the West of England, BS16 1QY, UK; b Biological, Biomedical and Analytical Sciences, University of the West of England, BS16 1QY, UK

**Keywords:** Microbial fuel cell (MFC) stack, Electricity production from urine, Ceramic membrane, Self-powered electronic faucet

## Abstract

Hygienic measures are extremely important to avoid the transmission of contagious viruses and diseases. The use of an electronic faucet increases the hygiene, encourages hand washing, avoids touching the faucet for opening and closing, and it saves water, since the faucet is automatically closed. The microbial fuel cell (MFC) technology has the capability to convert environmental waste into energy. The implementation of low cost ceramic MFCs into electronic interfaces integrated in toilets, would offer a compact powering system as well as an environmentally friendly small-scale treatment plant. In this work, the use of low cost ceramic MFCs to power an L20-E electronic faucet is presented for the first time. A single MFC was capable of powering an electronic faucet with an open/close cycle of 8.5 min, with 200 ml of urine. With a footprint of 360 cm^3^, the MFC could easily be integrated in a toilet. The possibility to power e-toilet components with MFCs offers a sustainable energy generation system. Other electronic components including an automatic flush, could potentially be powered by MFCs and contribute to the maintenance efficiency and hygiene of the public toilets, leading to a new generation of self-sustained energy recovering e-toilets.

## Highlights

• L20-E electronic faucet fully powered by a single Microbial fuel cell.
• Applicability of low cost-ceramic fuel cell treating urine.
• A single MFC with 200 ml of urine replaces the use of 4AA batteries.
• Towards self-sustained energy recovering e-toilets with the use of MFCs.

## Graphical abstract

**Figure:**
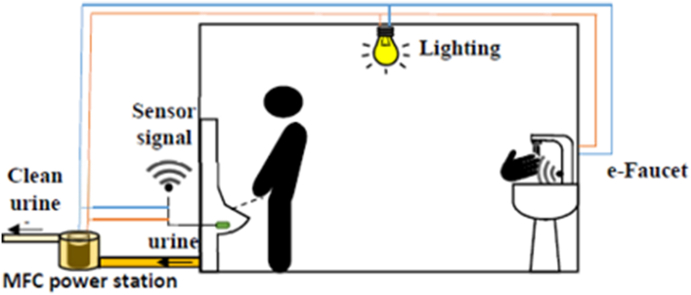

## 1 Introduction

The number of people living without electricity decreased to roughly 840 million between 2010 and 2019. Despite the notable progress made on energy access in recent years, the global energy targets set in the United Nations Sustainable Development Goals (SDG) to ensure affordable, reliable, sustainable and contemporary energy for all by 2030 are still far from being achieved. According to the International Energy Agency (IEA) the International Renewable Energy Agency (IRENA), the United Nations Statistics Division (UNSD), the World Bank and the World Health Organization (WHO) greater efforts are needed to reach this target. Particularly challenging is the improvement of the energy sustainability and the energy production in some of the world's poorest populations [1].

Microbial fuel cell (MFC) technology is an emerging sustainable energy technology, which produces electricity from human waste [2]. The combined advantages obtained from the MFC technology include energy recovery from waste, decentralised wastewater treatment, pathogen killing and electrochemical production of catholyte with disinfecting properties [[3], [4], [5], [6], [7], [8]]. These advantages together with the relatively new initiative of circular economy, which in itself requires new technological approaches for energy generation, waste management and resource recovery, position MFCs at the top of renewable energy technologies. The use of low cost materials such as ceramic membranes or carbon-based electrodes increases the possibility to implement such technology in the developing countries.

The practical implementation of a stack of microbial fuel cells generating electricity from wastewater has been reported in the past, where the energy generated under laboratory conditions was able to power a mobile phone, a wristwatch or an air freshener [8,9]. A real life implemented example was the use of ceramic MFCs in stacks to power the internal lighting of a toilet installed at the Glastonbury Music Festival at Worthy Farm, Pilton (UK) in June 2015 and fed from human urine donated by volunteers attending the festival [10]. An array of 12 modules containing 36 MFCs each, were connected in series and parallel to achieve the power demanded by the LED lighting. The urine was flowing in cascade mode passing through all the modules. The estimated number of users was ~1000 users/day and a COD reduction of 30% was achieved during the festival [10]. This field trial demonstrated the applicability of the MFC technology. Consecutive field trials to improve the technology and power LED lighting have been succeeding, including the installation of MFC stacks at the Glastonbury festival 2016, at a UWE Frenchay campus public urinal, and at the Sesame Girls School in the small village of Kisoro in Uganda [10,11].

The possibility of integrating the MFC technology in buildings was recently investigated by embedding the MFCs into conventional house bricks. This study suggests that the idea of converting existing and future buildings to micro-power stations and micro-treatment plants with the help of integrated MFCs and other renewable technologies, which will be a step closer to a truly sustainable life [12].

It is generally accepted in the scientific community that the energy density of an individual MFC unit is relatively low and the scale-up of several MFCs and large wastewater volumes are necessary to gain a meaningful power output for practical applications. However, recent findings can be implemented to maximise the MFC performance. Ieropoulos et al. [13] reported an increase in the power output profile from an MFC where the electrical load had been periodically switched off and then re-connected. This can be translated to an MFC performance increase, due to the relaxation and stress intermittency. According to this *modus operandi*, the MFC is stressed, when connected to an external load and then transiently relaxed, when the external load is removed (open circuit condition). When the external load is optimum for maximum power transfer, energy extraction from the MFC can be maximised and this can be easily exploited by pulse-width-modulation (PWM). If the energy generated is then harvested in a capacitor it can be stored and used when required. Similarly, an increase in performance has also been observed via galvanostatic discharge, in supercapacitive mode, where the MFCs are submitted to periodic current pulses, taking advantage of the accumulated charges at the electrochemical double layer formed at the electrodes of the MFC during the charge/discharge cycles [14].

In this work, a small stack of ceramic-based MFCs was developed to investigate whether as a collective (n = 6) or as a single individual MFC would be possible to repeatedly power an electronic faucet. The investigation was done in the context of autonomous smart bathrooms as well as communal toilets/wash rooms situated in remote, rural or off-grid environments. The objective was to ascertain whether the urine of those individuals using the toilet/wash-room facility would be sufficient to maintain reliable e-faucet operation for hand-wash. The longer term objective of this study is to establish whether the whole of the smart bathroom, including internet-of-things (IoT) smart sensors, can be powered by human waste, through the use of MFCs.

## 2 Materials and methods

A total of 6 individual MFCs were assembled with fine fire clay (FFC) ceramic cylinders of 84 mm height, an external diameter of 48 mm and 2.5 mm thickness, which acted as the membrane separation between anode and cathode and the MFC structure. Each MFC contained a 90 × 27 cm^2^ anode made of carbon veil material (20 g/m2, PRF Composites, Dorset, UK), which was then folded and wrapped around the ceramic cylinder. A stainless steel wire (0.5 mm, Scientific Wire Company) was used to hold the carbon veil anode tight to the ceramic membrane and acted as a current collector. The air breathing cathode electrode was introduced on the inside part of the ceramic cylinder, initially empty and open to air. The cathode electrodes were prepared as previously reported [15] by thoroughly mixing activated carbon powder (80 g, GBaldwin&Co, UK) with PTFE solution 20% (polytetrafluoroethylene, 60% wt. Sigma-Aldrich) and spreading the mixture on a carbon veil surface. The electrode and paste were then hot pressed. The cathode electrode was cut to 112 cm^2^ covering the inside area of the internal part of the ceramic cylinder.

The MFCs were initially inoculated with anaerobic activated sludge, obtained from the Wessex Water wastewater treatment plant in Saltford, UK, and mixed with urine (1:1 ratio) [16]. After three days of inoculation period, the MFCs were fed with 100% fresh urine collected anonymously from healthy volunteers, pooled together and stored in a 40 L collection tank (pH 8.9–9.2), at room temperature. Initially all the MFCs were electrically connected in parallel and individually fed with 200 ml of urine per day per MFC at a continuous flow rate of 9 ml h^−1^ giving a hydraulic retention time (HRT) of 22 h, which was retained throughout the experiment.

Polarisation experiments were performed by connecting the MFC to a decade box consisting of variable resistors D07 (ELC, France), with resistance values between 30 KΩ and 3.74 Ω, holding for 5 min each resistor. The anode and cathode polarisation were monitored with an Ag/AgCl reference electrode (1 M KCl, Sigma-Aldrich). The MFCs performance was individually monitored as well as the capacitor voltage by recording the voltage in volts (V) in time by using an Agilent data logging unit (KEYSIGHT, 34972A LXI data acquisition/Switch). Chemical oxygen demand (COD) was analysed by using the potassium dichromate oxidation method (COD HR test vials, Camlab, UK) with an MD 200 photometer (Lovibond, UK).

**Fig. 1:**
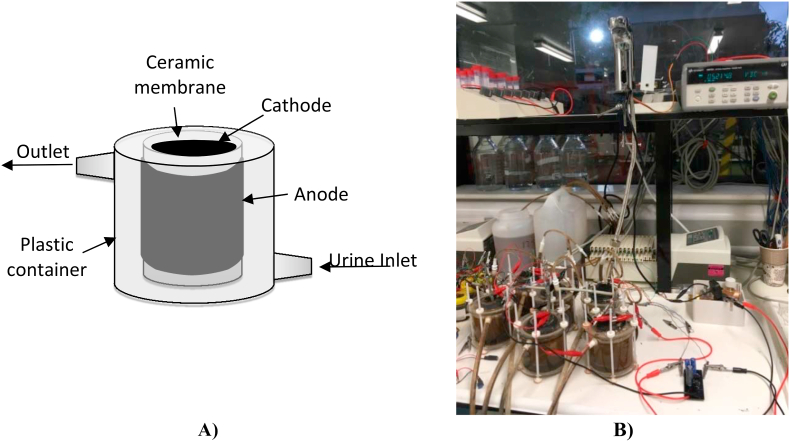
A) Schematic of the MFC assembly. B) Picture of the experimental set up when 6 MFCs were used to power the device.

The MFCs were then connected to an ultralow power energy harvester (SPV1050, ST life.augmented), which was connected to a storage capacitor, externally increased to 1 F and to the L20-E electronic faucet provided by ROCA Sanitario S.A. The technical characteristics of the faucet are listed in Fig. S1. A fixed resistor divider network was attached to the SPV1050 to set the MFCs to operate at their maximum power point, which was considered as 2/3 of their open circuit voltage. Due to the working voltage of the faucet, the output voltage was set from 4.2 V to 5.5 V. The diagram of the electronic connections is shown in Fig. S3. The electronic faucet had a stand-by consumption of 0.111 mW and a peak consumption of 2592 mW for 14.5 ms to open and close the electro-valve. This means an energy consumption per ON and OFF cycle of 80.4 mJ. The MFC charged the capacitor until the movement sensor activated the electronic faucet to be opened. An automatic system was set up to trigger the faucet every 8.5 min. Only the automatic system to trigger the electronic faucet was externally powered, since it simulates an individual (person) using the facilities. The experiment was initially performed with a stack of 6 MFCs connected to the electronic faucet. The number of MFCs connected to the stack was adjusted during the experiment, including tests of the e-faucet powered by 5, 4, 3 and 2 MFCs. Finally, the e-faucet was connected to a single MFC. Similarly, the frequency of the open/close cycle of the e-valve was also adjusted between 8.5 and 1 min to prove the MFC capability to power the faucet as often as required. Fig. 1 shows the schematic of the MFC assembly and the experimental set up when 6 MFCs were connected in parallel. A more detailed schematic diagram of the electrical connection of the MFCs in parallel to the faucet can be found in Fig. S2.

## 3 Results and discussion

**Fig. 2:**
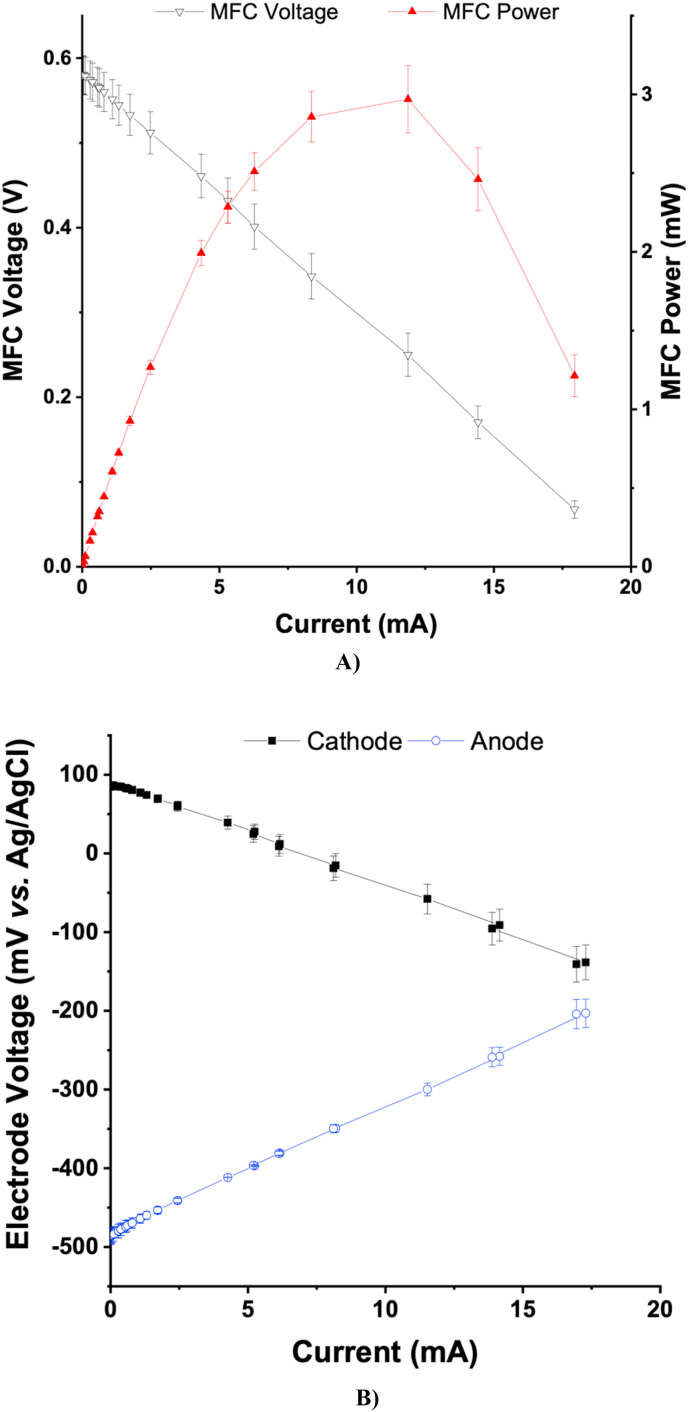
**A)** Average polarisation curve of the 6 MFCs, **B)** Anode and cathode polarisation versus Ag/AgCl reference electrode.

Fig. 2 shows the average levels from the polarisation curves of each individual MFC. The average open circuit voltage of the MFCs was 580 ± 22 mV and the average power generation was 3 ± 0.2 mW. To the authors knowledge the absolute power generated from the ceramic MFCs in this experiment is the highest obtained from fine fire clay ceramic MFCs compared to previously reported data [6,17,18]. As can be seen in Fig. 2B, the anode and cathode electrodes had optimum performance with none of them having significant limiting behaviour compared to each other.

Given that the main inoculum, from which the electroactive community grew, was anaerobic activated sludge and the only feedstock used was neat human urine, the anodic and cathodic reactions were the same as with similar previously reported work [27,28]. Besides treating urine and producing useful electricity, these ceramic MFCs have been reported to *in-situ* synthesise catholyte with disinfecting properties [[21], [22], [23], [24]]. The electricity generation from the MFCs has a high impact in the catholyte composition, obtaining a better quality catholyte when more electricity is generated. The physicochemical and bactericidal properties of the catholyte generated by this type of ceramic MFCs has been previously analysed, showing killing properties towards *E. coli* [23,24].

Once the polarisation curves demonstrated that the MFCs were operating under optimum conditions and providing maximum power output, they were connected to the electronic board and the e-faucet. Initially, all 6 MFCs were connected as a stack in parallel, maintaining the individual feeding with fresh urine. The MFC stack voltage and the capacitor voltage were monitored with time. The movement sensor was initially set up to trigger the electronic faucet every 1 min. It should be highlighted that the MFC stack, independently of the number of times the e-faucet was opened and closed, continuously powers the faucet movement sensor (0.251 mW). When the movement sensor triggers the faucet, the e-valve opens consuming a power peak of 2952 mW for 14.5 ms, which is provided by the capacitor connected to the MFCs.

**Fig. 3:**
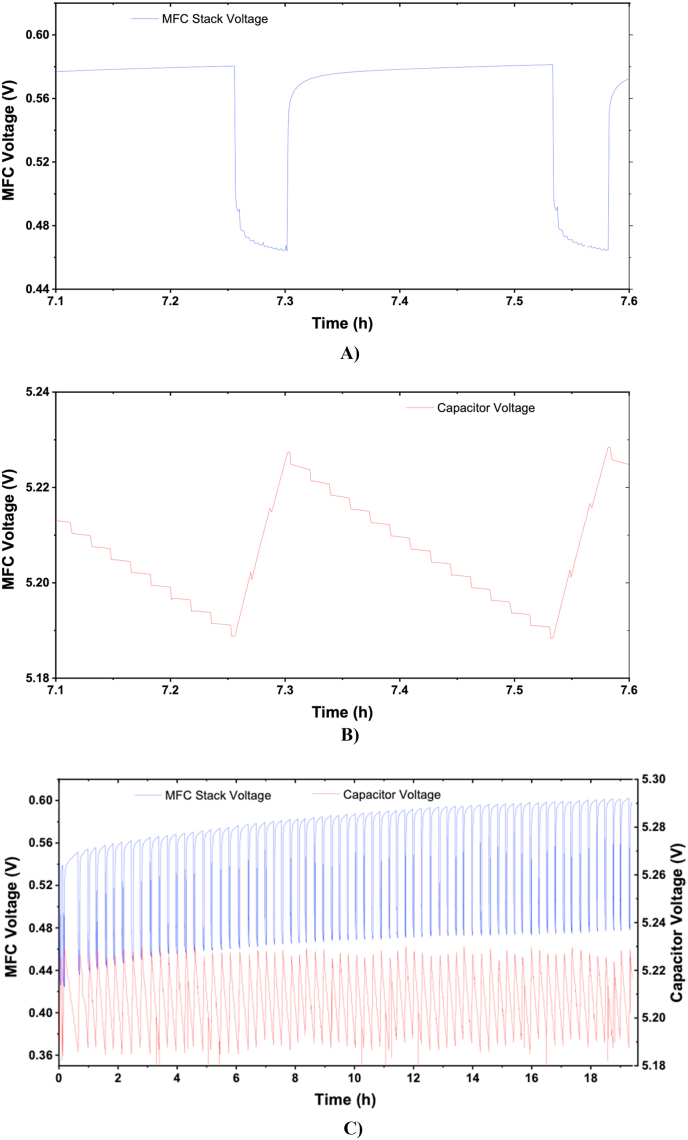
**A)** Results from 6 MFC powering the movement sensor and the electronic faucet opening and closing every 1 min, **B)** Charging/discharging cycle of the capacitor, C) Stress/relaxation cycles of the MFCs. Optimisation of the number of MFCs.

Fig. 3 shows the experimental results, where the charging/discharging cycles can be observed. According to Fig. 3, the results showed how the 6 MFCs could comfortably charge the movement sensor and the e-faucet valve triggered every 1 min. Fig. 3A) shows a detailed graph of the MFC stack voltage and the iterations of stress and relaxation period. When the MFC is connected to the electronic board, the MFC voltage initially drops (V_capacitive_) due to the discharge of the self-polarised electrodes and the non-faradaic current. After that, and considering that the MFCs are working under maximum power generation and therefore under an electron transfer limited regime, the voltage continues to decrease during the stabilisation period (V_ohmic_), until it reaches a steady voltage or until the load is disconnected from the MFC. This voltage drop is caused by the ohmic resistance of the electrodes and the electrolyte and the changes of substrate concentration at the electrode surface. When the MFC is under open circuit conditions, the concentration of substrate at the electrode surface is that of the bulk (*c*_*o*_). When the MFC is connected to the electronic board (stress period), and the bio-electrochemical reactions starts taking place, the concentration at the electrode is partially consumed until stabilised at a concentration *c*_*1*_ lower than the bulk [19]. During the relaxation period, the concentration of substrate at the electrode surface goes back to *c*_*o*_. By combining stress and relaxation periods, the system takes advantage of the accumulated charge due to the electrodes self-polarisation and the changes in concentration at the electrode surface from the bulk concentration *c*_*o*_ to the stabilised concentration *c*_*1*_.

Every time the movement sensor triggered the e-valve of the faucet to open and close, the capacitor was partially discharged and a small voltage drop can be observed (Fig. 3B). During the MFC relaxation period, the capacitor is discharging and its voltage decreases from approximately 5.22 V to approximately 5.19 V. When the faucet was closed, the MFC stack was charging the capacitor increasing its voltage from 5.19 V to 5.22 V. Once the capacitor was fully charged, the discharge cycle started, with the MFCs going into relaxation mode under open circuit conditions. This charging/discharging cycle was repeated periodically during the experiment.

As shown in Fig. 3C, the experiment was run for 20 h, in which the faucet was opened 1200 times. During this period, the MFC relaxation voltage increased from 0.55 V to 0.6 V during the experiment. This suggests that the MFC stack was not running under maximum power generation and that the MFC stack could provide more power than that required by the electronic board and the e-faucet. This indicated that the faucet could be triggered more often and that lower number of MFC units could be used to run the e-faucet.

**Fig. 4:**
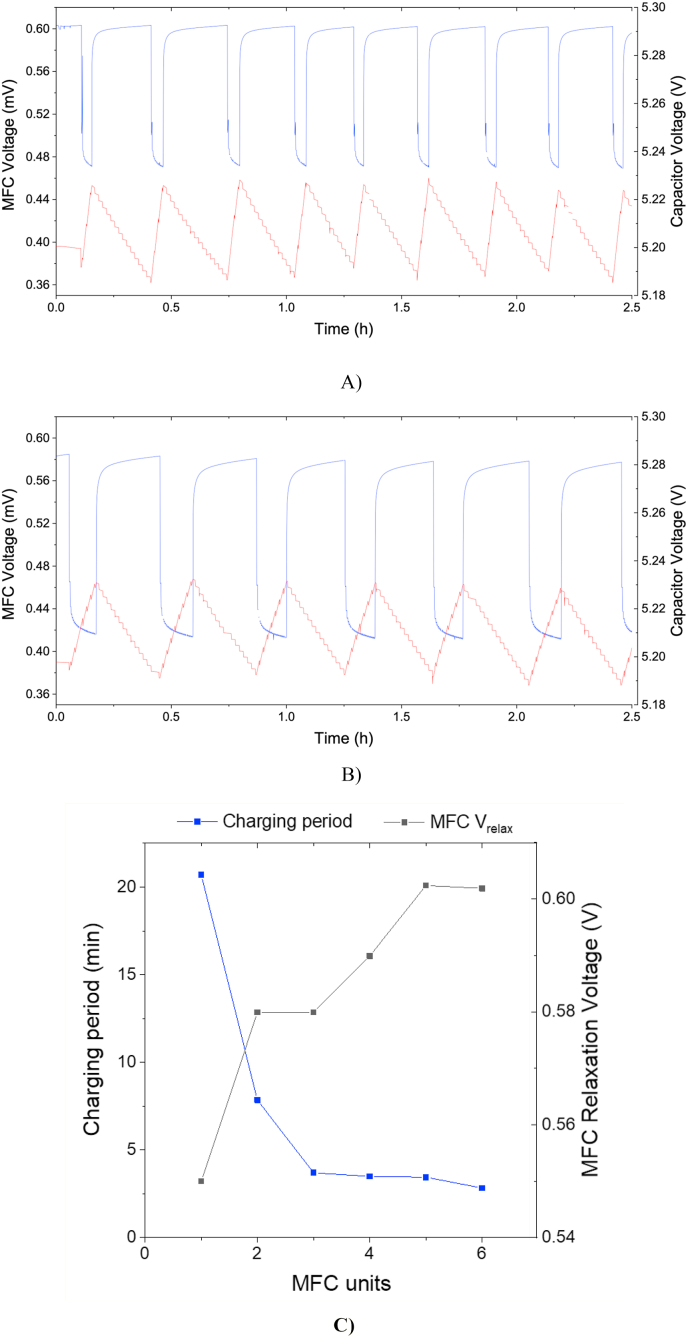
MFC voltage and capacitor charging/discharging cycles **A)** 5 MFC, **B)** 2 MFCs connected in parallel while powering the movement sensor and the electronic faucet opening and closing every 1 min. **C)** Summary of charging period and relaxation time.

The number of MFCs electrically connected to the electronic board was then sequentially reduced to 5, 4, 3 and 2 with no detriment to performance. Fig. 4A and B shows the fuel cell performance and the capacitor voltage when connecting 5 and 2 MFCs, respectively. Similar graphs corresponding to 4 and 3 MFCs can be found in Fig. S4. In these experiments, the faucet was opened/closed every 1 min. Fig. 4C shows the comparison of the charging and relaxation periods for different number of units connected in a stack. The capacitor charging time was shorter for a higher number of MFCs connected in parallel, as it was expected since higher power is contributing to the charging cycle. On the contrary, the capacitor discharging time remained constant (15 min) for a constant number of open/close cycles (every 1 min). It can also be observed that the MFC relaxation voltage and the MFC stress voltage reached lower values when fewer MFCs were connected as a stack to power the faucet, since the energy requirements remained constant for a lower number of MFC units in the stack.

### 3.1 E-faucet powered by a single ceramic MFC

**Fig. 5:**
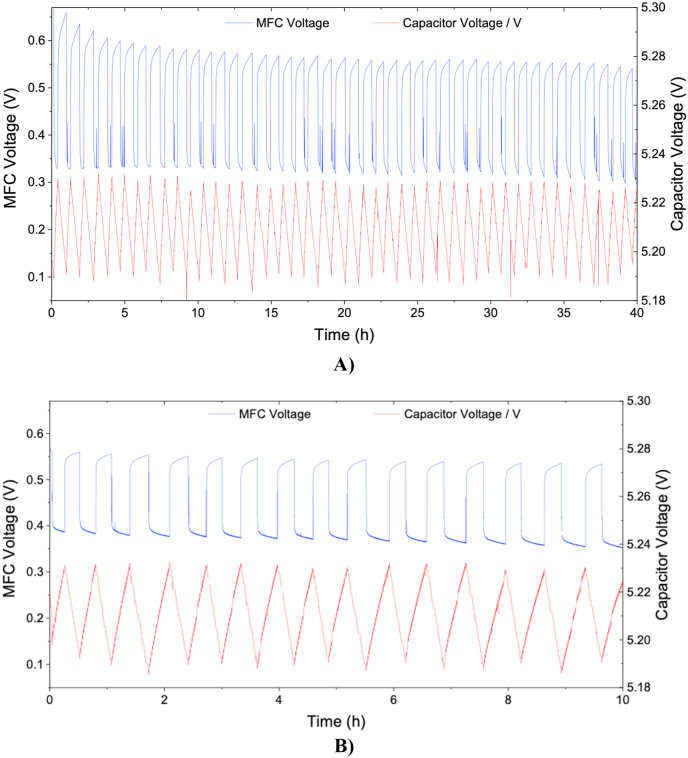
Single MFC voltage and capacitor voltage when only 1 MFC was connected to the electronic board and the e-faucet, which opened every. **A)** 3 min, **B)** 1 min.

Fig. 5 shows the stress/relaxation voltage of a single MFC and the charging/discharging cycles of the capacitor for open/close cycles or 1 and 3 min. A similar graph for 2 min is shown in Fig. S5. The MFC was being fed with 200 ml of urine per day. As shown in Fig. 5A) the single ceramic MFC was capable of powering the e-faucet for 40 h with open/close cycles every 3 min. The relaxation voltage decreased from 0.66 V after the first cycle to 0.54 V after 40 h of cycles. This decrease indicates that the MFC is operating under maximum power transfer conditions, and the relaxation period is not long enough to reach open circuit conditions. A similar trend can be observed in Fig. 5B) and C), where the MFC relaxation and stress voltages tend to decrease in time and for shorter open/close cycles. This decrease however, could be avoided by increasing the feeding rate of the MFC with a larger volume of fresh urine. In any case, these cycles are overestimating the MFC stress periods, since they consider that the movement sensor will be activated once every minute continuously for several hours. In the real world, the person will urinate and wash their hands and the sensor will not activate until the next user uses the facilities. Moreover, the toilet will not be used as often during night periods.

The MFCs used in this study were made from ceramic material (fine fire clay) and used carbon electrodes along with stainless steel wires to complete the setup. Although the manufacturing costs for the ceramic separator/MFC structural materials could not be accurately calculated, due to their small quantity, based on the cost of electrode and wire material previously calculated [26] it is valid to assume that the total cost of the fully assembled MFCs used in this study would be less than £1 per MFC unit. This is still high from a mass manufacturing perspective, but relatively low in lieu of this being a laboratory prototype.

### 3.2 Long-term operation

**Fig. 6:**
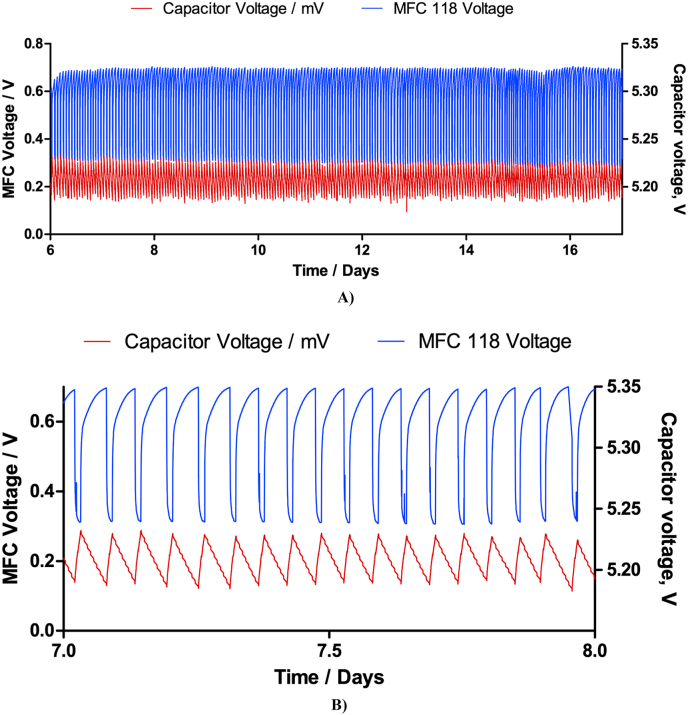
**A)** Results from only 1 MFC powering the movement sensor and the electronic faucet opening and closing every 8.5 min, **B)** Magnification from the green area in Fig. 2A showing the MFC voltage iterations and the charging cycle of the capacitor. (For interpretation of the references to colour in this figure legend, the reader is referred to the Web version of this article.)

Fig. 6 shows the results of 1 MFC fed with 200 ml of fresh urine per day (less than the individual production per visit to the toilet), being able to constantly power the movement sensor and the opening and closing valves from the faucet every 8.5 min. That is the equivalent to 169 open/close cycles per day. This value was chosen considering the estimated lifetime of the 4 AA batteries, which are generally used to power the e-faucet and should be able to open/close the e-faucet 150 times per day during 2 years. As shown in Fig. 6, the single MFC was comfortably repeating the charging/discharging cycles during 11 days, after which time the experiment was stopped. During the relaxation period, the MFC reached voltages of up to 0.74 V. This constitutes cell relaxation, which is an element that will be further exploited for wider use.

**Fig. 7:**
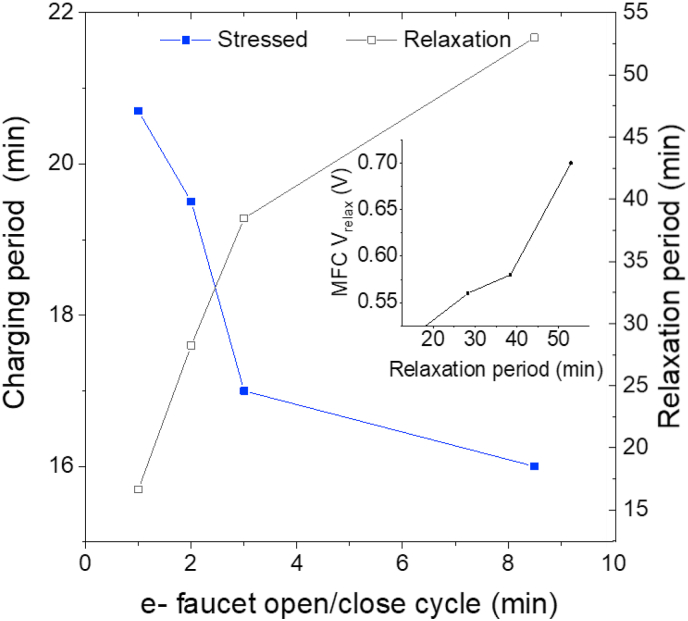
Average stress (left y-axis) and relaxation periods (right y-axis) versus the opening/closing cycles when using a single ceramic MFC for the charging of the electronic faucet. Inset: Changes of the MFC relaxation voltage with the relaxation period.

Fig. 7 shows a summary of the average stress and relaxation periods needed for the different open/close cycling times of the e-faucet powered by a single ceramic MFC. As expected, the MFC requires longer charging periods to power the e-faucet more often, whereas relaxation period increases with the MFC relaxation voltage reached.

### 3.3 Urine treatment

The COD reduction was on average over 14.5% per single MFC. When the 6 MFCs were in cascade a COD reduction of 70% was achieved. The initial COD concentration in urine 8100 ± 400 mg L^−1^ was reduced to 2430 mg L^−1^ and that each MFC is contributing more than 10% decrease in the COD. The addition of three more MFCs to the cascade, could potentially achieve a COD reduction of over 95% and the maximum legal discharge concentration, which according to the European Union is 125 mg COD·L^−1^ [25].

### 3.4 Future perspective

Nowadays, especially under the recent COVID pandemic, appropriate hygiene measures are even more important to help avoid the transmission of contagious viruses and diseases, the use of a touch-free electronic faucet is essential. Moreover, electronic faucets are more efficient in terms of water saving. The novelty described in this study is that no power supply is required other than the urine excreted by the user. The possibility of powering an e-toilet or smart toilet, with MFCs feeding on human waste, becomes more feasible, offering a sustainable energy generation system.

This experiment demonstrates a practical application of the MFCs fed with urine. However, other similar applications could be used, including powering of an electronic flushing urinal, electronic toilets (e-toilets) or sensor networks as part of IoT. E-toilets can be incorporated in developed and developing countries, improving the public health, hygiene, and sanitation sector. They can incorporate automatic light switching, toilet flushing or door opening.

The use of smart toilets could also improve the conditions in emergency settings or camps, achieving improvements in terms of cost savings, water saving, better services, and a vision for sustainability. The use of toilets equipped with sensors and information & communication technologies (ICT) such as the eSOS (emergency Sanitation Operation System) Smart Toilet®, for an efficient operation in an emergency setting, has been reported and tested in field trials with successful results [20]. Moreover, the addition of remote monitoring capabilities can facilitate public health state tracking by using sensing elements and electronic systems embedded into the toilets. The integration of the MFC technology to power these types of sensor and ICT approaches can also provide a sustainable solution and clean energy to emergency situations, where the lack of energy is a significant problem in itself.

Moreover, ceramic MFCs can synthesise disinfecting catholyte *in situ* and treat the urine to legally dischargeable levels. These are added value elements that contribute to an improved sanitation, facilitates personal hygiene, decreasing the risk of water-borne diseases and the spread of infectious diseases.

## 4 Conclusions

This work demonstrates a functional application of the low cost ceramic MFCs. A single ceramic MFC unit with a footprint of 84 mm height and 48 mm external diameter and 200 ml of urine is capable to power a movement sensor continuously and an electronic valve to open/close an electronic tap 169 times per day for 11 days. This is the equivalent of open/close cycles every 8.5 min. This offers a robust, simple and direct retrofit system to permanently replace the use of the 4 AA battery required to power the electronic faucet, with no need for periodic replacement every two years, which is the estimated lifetime of the batteries considering 150 cycles of opening and closing the electro-valve of the faucet per day (~8.5 min interval). Moreover, this system offers a simultaneous decentralised wastewater (in this case urine) treatment. The MFC system presented here takes advantage of the relaxation intermittency to increase the microbial fuel cell performance and to make use of the capacitive energy accumulated during the relaxation period.

This work presents a robust, simple and directly implementable technology for urine treatment and electricity generation from urine to power an L20-E electronic faucet provided by ROCA sanitary, Spain. Moreover, this work demonstrates the applicability of the MFC technology to improve hygiene and sanitation, working autonomously and independent of the energy grid, providing that there is running clean water.

## CRediT authorship contribution statement

**Irene Merino Jimenez:** Writing – review & editing, Data curation, Investigation. **Patrick Brinson:** Writing – review & editing, Data curation, Investigation. **John Greenman:** Conceptualization, Supervision. **Ioannis Ieropoulos:** Writing – review & editing, Conceptualization, Supervision, Project administration, Funding acquisition.

## Declaration of competing interest

The authors declare that they have no known competing financial interests or personal relationships that could have appeared to influence the work reported in this paper.
