# Supplementary material for: Electronic faucet powered by low cost ceramic microbial fuel cells treating urine
Source: J Power Sources. 2021 Sep 15;506:230004. doi: 10.1016/j.jpowsour.2021.230004 (PMC8363936; doi:10.1016/j.jpowsour.2021.230004)
Supplement: Multimedia component 1 [file mmc1.docx]

**Supplementary Information of the paper:**

**Electronic faucet powered by low cost ceramic microbial fuel cells treating urine**

Irene Merino Jimenez^a^, Patrick Brinson^a^, John Greenman^a,b^, Ioannis Ieropoulos^a,b^

^a^ Bristol BioEnergy Centre, Bristol Robotics Laboratory, University of the West of England, BS16 1QY, UK

^b^ Biological, Biomedical and Analytical Sciences, University of the West of England, BS16 1QY, UK

Figure S1. Specifications of the power requirements for the L20-E faucet provided by ROCA Sanitarios S.A.

| 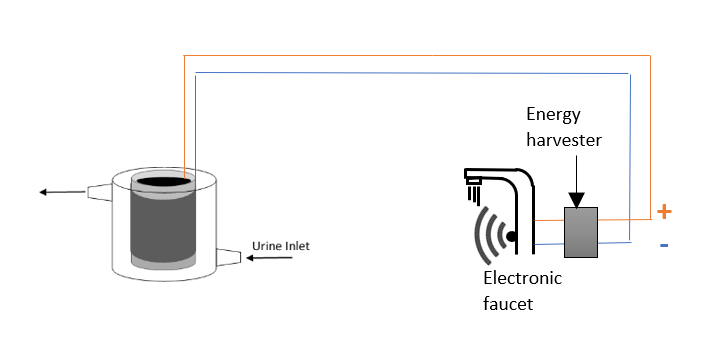  A) |
| --- |
| 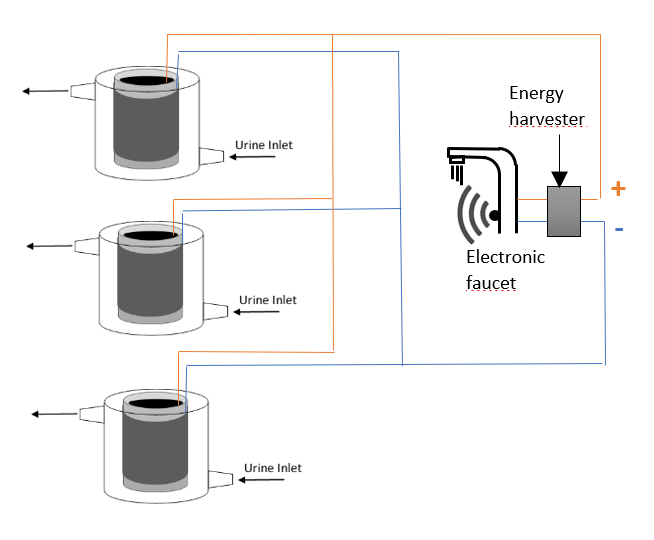  B) |

Figure S2. Schematic of: A) a single MFC connected to the electronic faucet, B) three MFCs connected in series to the electronic faucet and individually fed.


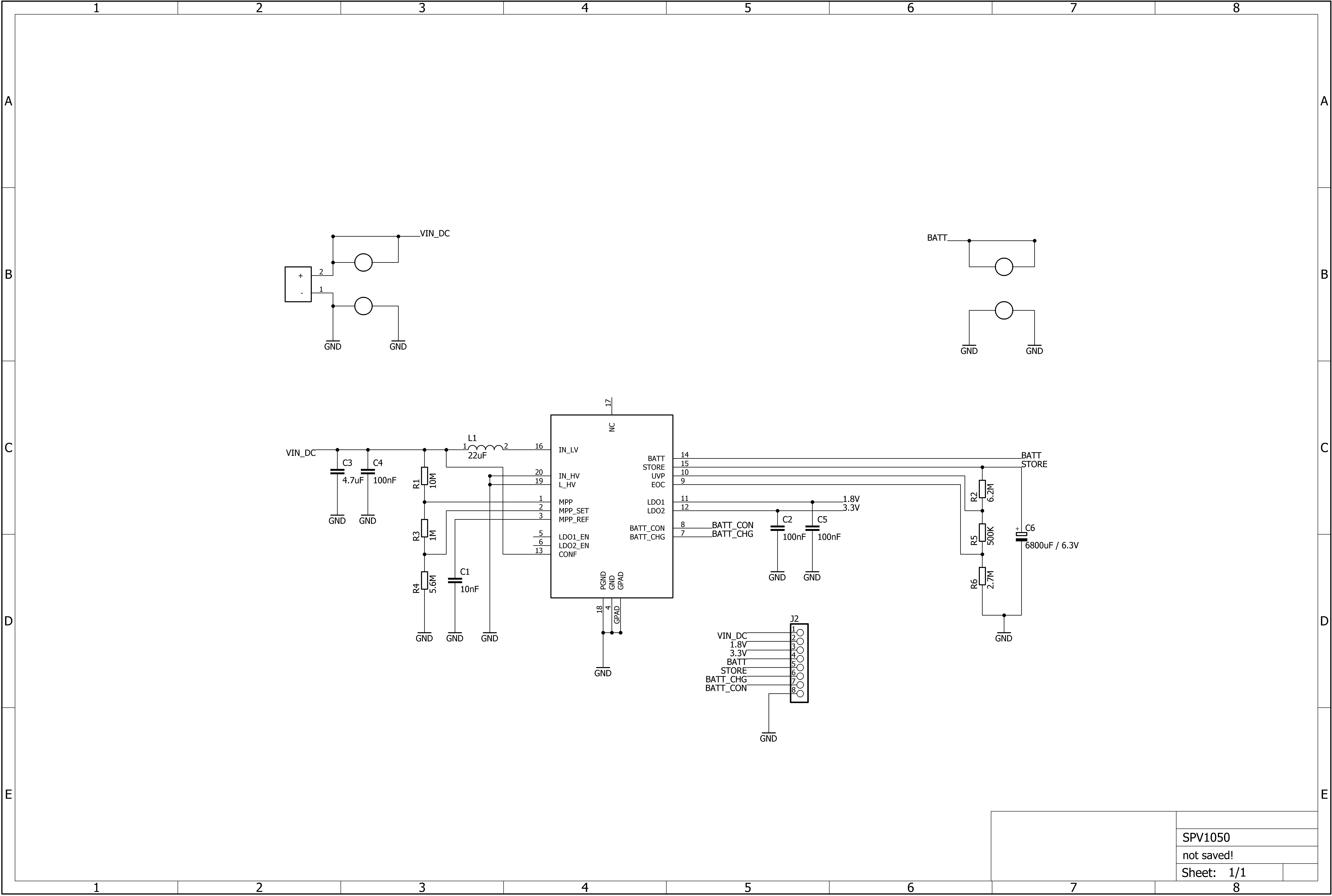


Figure S3. Diagram of the electronic components connected to the MFC technology and the electronic faucet.

|   A) |
| --- |
|   B) |

**Figure S4.** MFC voltage and capacitor charging/discharging cycles **A)** 4 MFCs, **B)** 3 MFCs connected in parallel while powering the movement sensor and the electronic faucet opening and closing every 1 minute.

**Figure S5.** Single MFC voltage and capacitor voltage when only 1 MFC was connected to the electronic board and the e-faucet, which opened every 2 minutes.
